# Genome Sequences of Foot-and-Mouth Disease Viruses of Serotype O Lineages Mya-98 and Ind-2001d Isolated from Cattle and Buffalo in Myanmar

**DOI:** 10.1128/MRA.01737-18

**Published:** 2019-02-21

**Authors:** Wint Yi Maung, Tatsuya Nishi, Tomoko Kato, Khin Ohnmar Lwin, Katsuhiko Fukai

**Affiliations:** a Livestock Breeding and Veterinary Department, BSL-2 Foot-and-Mouth Disease Diagnostic Laboratory, Shwemyo, Naypyidaw, Myanmar; b Exotic Disease Research Unit, Division of Transboundary Animal Diseases, National Institute of Animal Health, National Agriculture and Food Research Organization, Kodaira, Tokyo, Japan; c Ministry of Agriculture, Livestock and Irrigation, Naypyidaw, Myanmar; Loyola University Chicago

## Abstract

We report whole-genome sequences and partial genome sequences of eight foot-and-mouth disease viruses obtained from outbreak samples in Myanmar in 2016 and 2017. These viruses are classified into O/ME-SA/Ind-2001d and O/SEA/Mya-98 lineages.

## ANNOUNCEMENT

Foot-and-mouth disease (FMD) is endemic in Asia, the Middle East, and Africa (1, 2). The FMD virus (FMDV) genome is a single strand of positive-sense RNA and over 8,000 bases in length. FMDV is divided into seven serotypes, namely, O, A, C, Asia1, and Southern African Territories 1 to 3. Each serotype is divided into several topotypes and lineages by genetic characterization (3).

Serotype O is the most frequently detected, and three topotypes exist in Southeast Asia, namely, Southeast Asia (SEA), Cathay, and Middle East-South Asia (ME-SA) topotypes (4, 5). Ind-2001 is a lineage of serotype O, ME-SA topotype, and has been dominant in the Indian subcontinent since 2008; however, it was rare in Southeast Asia (6). In 2015, Ind-2001d was detected first in Vietnam and Laos (7). In 2016, Ind-2001d was detected in Myanmar (8, 9).

Eight epithelial samples were collected from cattle and a buffalo that showed clinical symptoms of FMD in Myanmar in 2016 and 2017. Viruses were passaged using the ZZ-R 127 cells (10). Viruses were isolated from two samples collected in the Yangon Region (O/MYA/Yan/3/2016 and O/MYA/Yan/5/2016). No viruses were isolated from the remaining samples. Viral RNAs were extracted using a High Pure viral RNA kit (Roche Diagnostics), and first-strand cDNA synthesis was performed using SuperScript III reverse transcriptase (Life Technologies) and two primers (11). RT-PCR and sequencing for the VP1 region were performed using the primers ARS4 (5′-ACCAACCTCCTTGATGTGGCT-3′) and NK61 (5′-GACATGTCCTCCTGCATCTG-3′). The whole viral genome was amplified by PrimeSTAR Max DNA polymerase (TaKaRa) and four primer sets (11). Viral whole-genome nucleotide sequences were determined using the Ion PGM system (Life Technologies) as previously described (11). Using Torrent suite software version 5 with default parameters (Life Technologies), 47,286 reads in total (7,765,015 bp) of O/MYA/Yan/3/2016 and 35,681 reads in total (6,476,019 bp) of O/MYA/Yan/5/2016 were assembled and mapped, with a depth of coverage of more than 10 reads. Final assemblies of O/MYA/Yan/3/2016 and O/MYA/Yan/5/2016 were 7,692 and 7,733 nucleotides (nt) in length, with 98.5% and 99.1% L-fragment sequence coverage, G+C content of 53.5%, and an average coverage depth of 945.7 and 788.7, respectively.

Sequences of the VP1 region (639 bp) of O/MYA/Bag/1/2016, O/MYA/Aye/2/2016, and O/MYA/Sag/10/2017 collected from cattle in the Bago, Ayeyarwaddy, and Sagaing regions, respectively, were most closely related to that of O/MYA/05/2009 (GenBank accession number KR401156), with a 93% nt identity rate and no indels based on NCBI BLAST analysis, and were classified into the O/SEA/Mya-98 lineage. O/SEA/Mya-98 FMDVs have been circulating in Myanmar for a long time, and viral genetic substitutions would be accumulated by selection pressure of transmission across hosts and immunity induced by infection or vaccination.

Sequences of the VP1 region (639 bp) of O/MYA/Yan/3/2016, O/MYA/Yan/5/2016, O/MYA/Aye/4/2016, O/MYA/Sag/7/2017, and O/MYA/Rak/8/2017 collected from cattle and a buffalo in the Yangon, Ayeyarwaddy, and Sagaing regions, and Rakhine State, respectively, were most closely related to that of O/BAN/GO/Ka-236(Pig)/2015 (GenBank accession number KX712091), with a 99% nt identity rate and no indels based on NCBI BLAST analysis, and were classified into the O/ME-SA/Ind-2001d lineage.

In conclusion, we report complete genome sequences of two O/ME-SA/Ind-2001d FMDVs and partial sequences of three O/ME-SA/Ind-2001d and three O/SEA/Mya-98 FMDVs. We need to monitor the epidemiological information of outbreaks contributing appropriate FMD control strategies.

### Data availability.

Nucleotide sequences of eight FMDVs have been deposited in GenBank under accession numbers LC438819 to LC438823 and LC439252 to LC439254. Raw sequence reads were deposited in the Sequence Read Archive (SRA) under BioProject accession number PRJDB7794 and SRA project accession number DRP004683 (O/MYA/Yan/3/2016), and BioProject accession number PRJDB7812 and SRA project accession number DRP004682 (O/MYA/Yan/5/2016).
